# A neural network-based method for polypharmacy side effects prediction

**DOI:** 10.1186/s12859-021-04298-y

**Published:** 2021-07-24

**Authors:** Raziyeh Masumshah, Rosa Aghdam, Changiz Eslahchi

**Affiliations:** 1 Department of Computer and Data Sciences, Faculty of Mathematical Sciences, Shahid Beheshti University, Tehran, Iran; 2 School of Biological Sciences, Institute for Research in Fundamental Sciences (IPM), Tehran, Iran

**Keywords:** Polypharmacy side effects prediction, Neural network, Drug–protein interactions, Drug–drug interactions

## Abstract

**Background:**

Polypharmacy is a type of treatment that involves the concurrent use of multiple medications. Drugs may interact when they are used simultaneously. So, understanding and mitigating polypharmacy side effects are critical for patient safety and health. Since the known polypharmacy side effects are rare and they are not detected in clinical trials, computational methods are developed to model polypharmacy side effects.

**Results:**

We propose a neural network-based method for polypharmacy side effects prediction (NNPS) by using novel feature vectors based on mono side effects, and drug–protein interaction information. The proposed method is fast and efficient which allows the investigation of large numbers of polypharmacy side effects. Our novelty is defining new feature vectors for drugs and combining them with a neural network architecture to apply for the context of polypharmacy side effects prediction. We compare NNPS on a benchmark dataset to predict 964 polypharmacy side effects against 5 well-established methods and show that NNPS achieves better results than the results of all 5 methods in terms of accuracy, complexity, and running time speed. NNPS outperforms about 9.2% in Area Under the Receiver-Operating Characteristic, 12.8% in Area Under the Precision–Recall Curve, 8.6% in F-score, 10.3% in Accuracy, and 18.7% in Matthews Correlation Coefficient with 5-fold cross-validation against the best algorithm among other well-established methods (Decagon method). Also, the running time of the Decagon method which is 15 days for one fold of cross-validation is reduced to 8 h by the NNPS method.

**Conclusions:**

The performance of NNPS is benchmarked against 5 well-known methods, Decagon, Concatenated drug features, Deep Walk, DEDICOM, and RESCAL, for 964 polypharmacy side effects. We adopt the 5-fold cross-validation for 50 iterations and use the average of the results to assess the performance of the NNPS method. The evaluation of the NNPS against five well-known methods, in terms of accuracy, complexity, and running time speed shows the performance of the presented method for an essential and challenging problem in pharmacology. Datasets and code for NNPS algorithm are freely accessible at https://github.com/raziyehmasumshah/NNPS.

**Supplementary Information:**

The online version contains supplementary material available at 10.1186/s12859-021-04298-y.

## Introduction

Drug combination, commonly referred to as polypharmacy, has become a common practice in modern medicine especially in elderly and patients with complex diseases [1–9]. While this strategy may treat the diseases more effectively, drug-drug interactions (DDIs) can occur unexpectedly [5, 6, 10–18]. DDI is a change in the pharmacologic effect of one drug when used with another drug. DDIs are the most common reason for patients to go to emergency units [4, 6, 12, 19–22] and can associate with Adverse Drug Reactions (ADRs) (i.e. side effects) including death, and it is a critical problem for public health [6, 10, 23–27]. Shtar et al. demonstrated that between 3 and 5% of all hospital medication injuries were dedicated to DDI [19]. Although some side effects can be discovered in experiments and clinical trials, they are usually costly and consuming time [10]. Most of the known polypharmacy side effects are rare and they are usually not observed in small clinical trials. So, it is difficult to identify these side effects manually [16]. Therefore, developing computational methods is desired for predicting DDIs. The methods in DDI prediction problem are divided into two categories. The first category just determines the presence or the absence of interactions, and they do not detect the type of side effects. These methods collect the interactions via experiments and clinical studies, medical records, and also through network modeling based on DDIs similarities, side effects similarities, and structure similarities [11, 28–41]. On the other hand, the goal of the second category is determining the type of side effects between drugs [16, 42–45]. To reduce the impact of polypharmacy side effects, the methods in the second category execute their role. In the following, some studies are expressed which address this issue. Nickel et al. proposed the relational learning approach named RESCAL which was based on a tensor factorization method [42]. DEDICOM was introduced by Papalexakis et al. and similar to RESCAL method was based on tensor decomposition [43]. Deep Walk method was based on a neural embedding approach which used a logistic regression classifier [44, 45]. The concatenated drug features method used a gradient boosting trees classifier to predict side effects [16]. Zitnik et al. designed a multi-relational method called Decagon, which was based on a tensor factorization decoder [16]. In this study, we develop neural network-based method for polypharmacy side effects prediction (NNPS). NNPS utilizes the neural network model mentioned with novel features achieves better results in comparison with the results of 5 well-known methods in terms of accuracy, complexity, and running time speed.

In next section, we describe the required datasets and the details of NNPS algorithm. In results section, the results of the NNPS model are compared with the results of the Decagon, Concatenated drug features, Deep Walk, DEDICOM, and RESCAL methods. The conclusion and some possible further works are presented in Discussion Section.

## Method

### Datasets

In this section, the mono side effects, the drug–protein interactions (DPIs), and the DDIs information are presented in details. In the following, we describe the databases and the summary of these databases is given in Table 1.

**Table 1:** Databases details

Matrix	Databases	Details of databases	Details of matrix	Ref
DDIs	TWOSIDES	No. drugs = 645 No. pairs = 63,473 No. side effects = 1317	No. drugs = 645 No. pairs = 63,473 No. side effects = 964	[46]
Mono side effects	SIDER OFFSIDES	No. drugs = 1556 No. side effects = 5868 No. drugs = 1332 No. side effects = 10,097	No. drugs = 645 No. side effects = 10,184	[46, 47]
DPIs	STITCH	No. CC = 519,022 No. proteins = 8934 No. int = 8,083,600	No. drugs =645 No. proteins = 8934 No. int = 18,690	[48–51]

*DDIs* Drug–drug interactions, *DPIs* Drug–protein interactions, *CC* chemical compound, *int* interaction

#### Drug–drug interactions and mono side effects information

As the multi-drug treatment is a common way [1–3], and modification in drug effect by another drug which is called DDIs, can produce adverse side effects, so, the knowledge of side effects information of DDI becomes the key issue in drug development and disease treatment. The DDI side effects (polypharmacy side effects) are collected from the TWOSIDES database [46]. TWOSIDES provides a reliable and comprehensive database for DDIs and has 1317 side effects on 645 drugs across 63,473 drug pairs. TWOSIDES is extracted from the Food and Drug Administration (FDA) Adverse Event Reporting System (FAERS). Like the previous study in the predicting polypharmacy side effects task [16], we consider 964 polypharmacy side effects which are occurred in at least 500 DDIs.

The side effects of individual drugs (mono side effects) are obtained from Side Effect Resource (SIDER) and OFFSIDES databases [46, 47]. The information of SIDER database is extracted from drug labels and contains 1556 drugs and 5868 side effects compiled from public documents. The information of OFFSIDES database is observed during clinical trials and contains 1332 drugs and 10,097 off-label side effects. Like TWOSIDES, OFFSIDES was generated from FAERS that collected from doctor reports, patients, and drug companies. Finally, by the union and the elimination of synonym side effects in SIDER and OFFSIDES databases, for 645 drugs which are in TWOSIDES database, 10,184 mono side effects are obtained.

#### Drug–protein interactions

DPIs are obtained from the Search Tool for Interactions of Chemicals (STITCH) database, which provide relationships between drugs and target proteins [48–51]. By using the STITCH database, we gain interactions between 8934 proteins and 645 drugs which are in TWOSIDES database. The number of interactions between these proteins and drugs is 18,690.

### Feature vectors

**Fig. 1:**
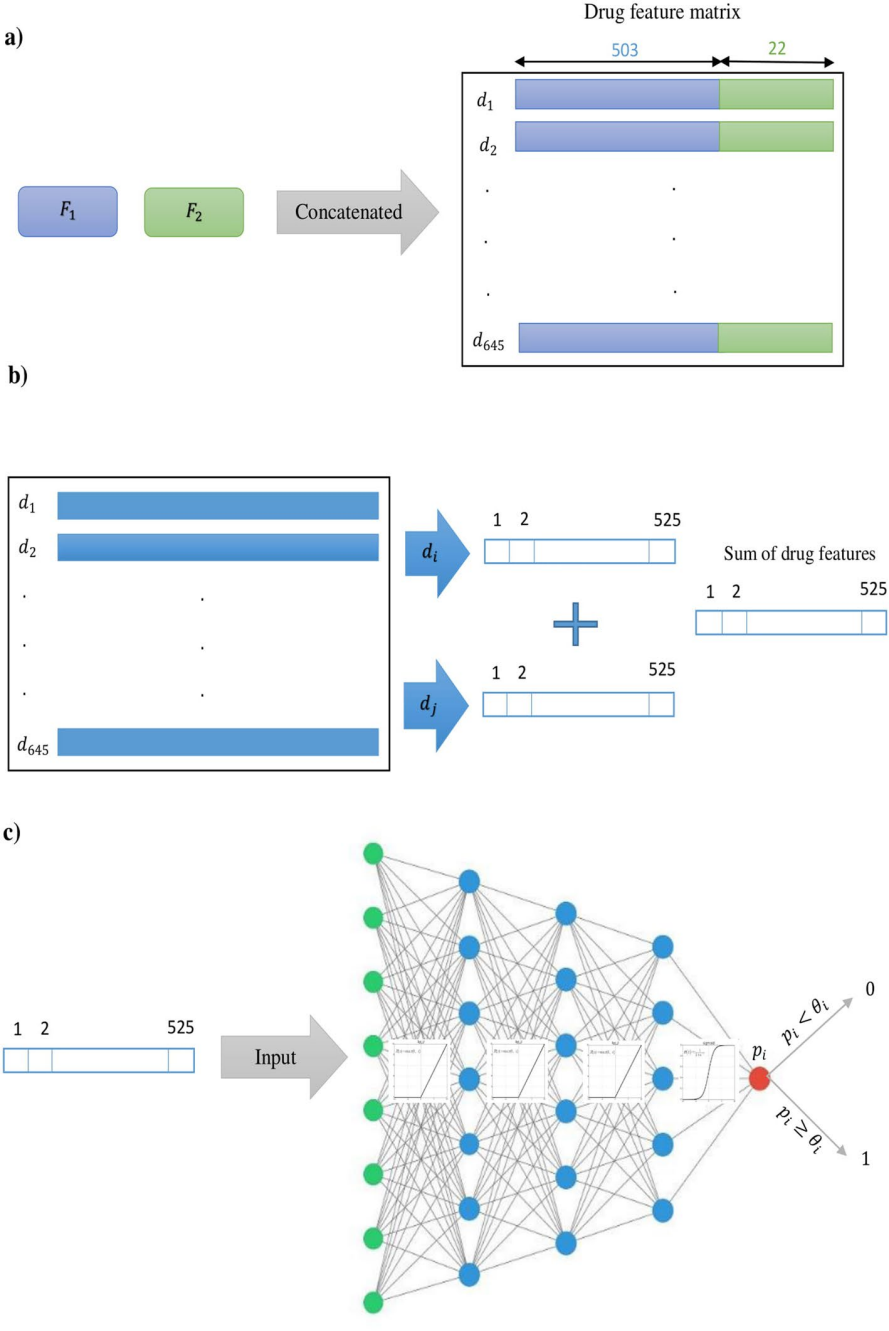
For the i-th side effect, the NNPS architecture is used. **a** Concatenation of the PCA representation of mono side effects $(F_{1})$ (blue) and the PCA representation of drug–protein interactions $(F_{2})$ (green). **b** Sum of the i-th and j-th rows in the drug features matrix for each $d_{i}$ and $d_{j}$ drug pair. **c** A three-layer neural network that computes the probability $p_{i}$ and classifies the i-th side effect based on the threshold i

For each side effect, two types of feature matrices including mono side effects matrix with dimension $645 \times 10{,}184$ and DPIs matrix with dimension $645 \times 8934$ are considered. Due to the large length of the features and their sparsities, using the feature extraction methods can be an effective way to reduce the size of features without losing important information. So, the Principle Components Analysis (PCA) is applied on mono side effects and DPIs matrices. The minimum number of the principle components is chosen such that 95% on variance in each matrix is retained. Two reduced feature matrices are denoted by $F_{1}$ with dimension $645 \times 503$ and $F_{2}$ with dimension $645 \times 22$, respectively. Then, by concatenating $F_{1}$ (blue) and $F_{2}$ (green), the drug feature matrix with dimension $645 \times 525$ is resulted (Fig. 1a). The rows of the resulting drug feature matrix indicate the drugs ID, while the columns show the features information. For a given drug pair $(d_{i}, d_{j})$, i-th and j-th rows of the drug feature matrix are summed for representing the drug-drug pairs feature and feed to the neural network (Fig. 1b).

### Training the neural network model

The drug pairs associated with each type of side effects are split into training, validation, and test sets, and 5-fold cross-validation is considered. We use 80 percent of drug pairs for the training set, 10 percent for the validation set, and 10 percent for the test set. The following steps are considered to achieve the best neural network architecture based on training datasets.

1. The number of hidden layers: $\lbrace 1,2,3,4,5 \rbrace$
2. The number of neurons in hidden layers: $\lbrace 25,50,100,200,300 \rbrace$
3. Activation functions: $\lbrace$Rectified Linear Unit (ReLU), hyperbolic tangent (tanh), and sigmoid$\rbrace$
4. The dropout rate: $\lbrace 0.1,0.3,0.5 \rbrace$
5. The learning rate: $\lbrace 0.01,0.001 \rbrace$
6. The momentum: $$\lbrace 0.7,0.9 \rbrace$$

We trained several networks with two, three, four, and five hidden layers and varying numbers of neurons (300, 200, 100, 50 and 25). We have included the best results for each trained network in the Table 2. As shown in this table, training a network with three hidden layers improves the results without significantly increasing the training time when compared to training a network with two hidden layers. The results improve slightly for networks with four or five hidden layers, but the computational time increases significantly. We chose a network with three hidden layers with 300, 200, and 100 neurons, respectively, due to the significant increase in computational cost and little benefit in terms of model performance of other structures. We had good results in terms of both Area Under the Receiver-Operating Characteristic (AUROC) and Area Under the Precision–Recall Curve (AUPRC) for the mentioned network, with a computational time of 8 h and 40 min.

**Table 2:** Results of different neural network architectures

No. hidden layers	No. neurons	AUROC	AUPRC	Running time
2	300, 200	0.961	0.950	8 h, 10 min
3	300, 200, 100	**0.966**	**0.953**	**8 h, 40 min**
4	300, 200, 100, 50	0.966	0.954	8 h, 50 min
5	300, 200, 100, 50, 25	0.967	0.954	14 h

The selected model is indicated in bold

### The architecture of neural network

**Fig. 2:**
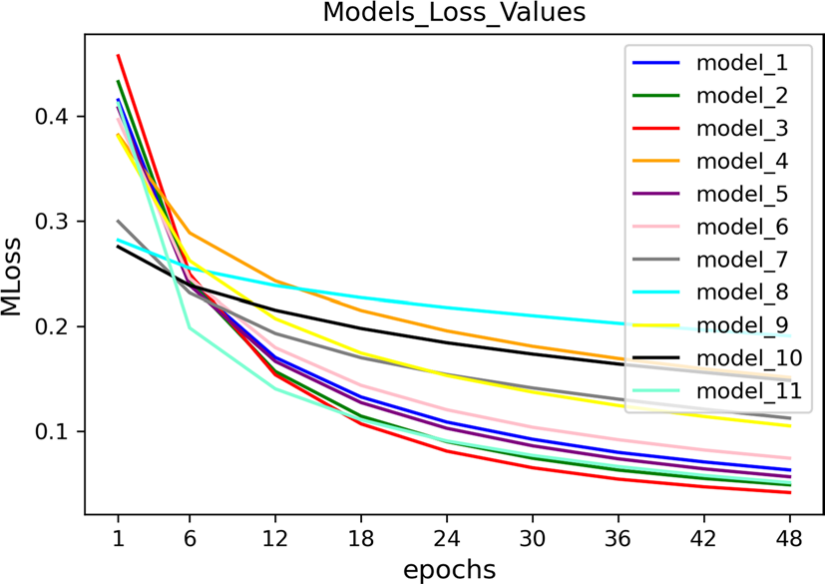
Loss curves of models based on different parameters for 50 epochs over all 964 polypharmacy side effects

The Neural Network is a feedforward network with fully connected layers consisting of an input layer, three hidden layers, and the output layer (Fig. 1c). The number of input layer neurons is equal to the size of the feature vector with size 525. The output layer has one neuron with probability value. For i-th side effect, we assign a class 0 (absence an interaction) or 1 (represent an interaction) to the output by using a threshold $\theta _{i}$ in the range of (0, 1). If the probability value is greater than $\theta _{i}$, the method suggests that the i-th side effect represents in the selected pair of drugs, otherwise, this side effects is not represent in the considered pair of drugs. For initialization weights, the Glorot normal initializer, also called Xavier normal initializer is applied [52]. By learning and investigating the results of the activation function of the neural network, we utilize the ReLU activation function between the layers of the neural model and consider a sigmoid activation function for the output layer (Fig. 1c). The optimization of the model parameters is done by using the binary-cross-entropy loss function and Stochastic Gradient Descent (SGD) [53]. In addition, we trained datasets based on different parameters (see Additional file 1: Table S1). We calculated and averaged loss value (MLoss) of each model over all 964 side effects for each epoch. Figure 2 shows the results of this investigation. In this work, MLoss is obtained by the following formula:

1 $$\begin{aligned} MLoss_{i} =\frac{\Sigma _{j=1}^{964}Loss_{side~effect_{j}}}{964} ,\quad for\;epoch\; i=1,\ldots ,50 \end{aligned}$$

**Fig. 3:**
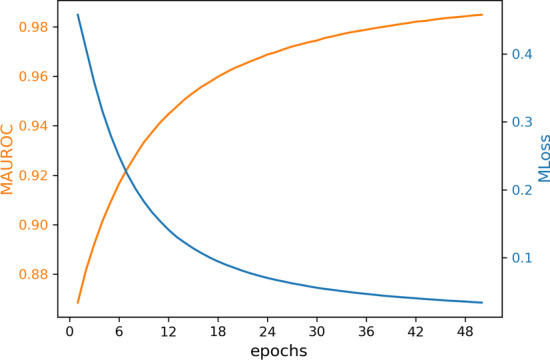
MAUROC and MLoss of NNPS model for 50 epochs over all 964 polypharmacy side effects

We depicted the Fig. 3 that considered AUROC against loss value when selecting epoch for the best performing model (NNPS).To do so, we calculated and averaged the AUROC (MAUROC) and MLoss of the best performing model for each epoch over all 964 side effects and plotted them, where MAUROC is obtained by the following formula:

2 $$\begin{aligned} MAUROC_{i} =\frac{\Sigma _{j=1}^{964}AUROC_{side~effect_{j}}}{964} ,\quad for\;epoch\; i=1,\ldots ,50 \end{aligned}$$

As shown in this figure, the considering structure works well across 964 polypharmacy side effects. As a result, we considered epoch 50 based on Figs. 2 and 3 for the best performance model of our neural network.

## Results

### Training hyperparameters

According to Fig. 2, the hyperparameters based on 5-fold cross-validation for the best model which we named NNPS are tuned by 50 epochs and batch size 1024 with a dropout rate of 0.1 for preventing over-fitting and learning rate 0.01 and momentum value 0.9 by trial and error are considered. Because the presence or absence of polypharmacy side effects is determined by a threshold, a ROC curve for each side effect is plotted, and the threshold $\theta _{i}$ with the highest F-score value is chosen. The hyperparameter values, the standard deviation, and the average thresholds for NNPS method are shown in Table 3.

**Table 3:** The selected hyperparameter values

No. hidden layers	No. neurons	Dropout rate	Learning rate	Momentum	SD $\theta _{i}$	Mean $\theta _{i}$
3	300, 200, 100	0.1	0.01	0.9	0.166	0.530

Threshold for 964 side effect ($\theta _{i}$), Standard Deviation (SD)

### Assessment and comparison

**Fig. 4:**
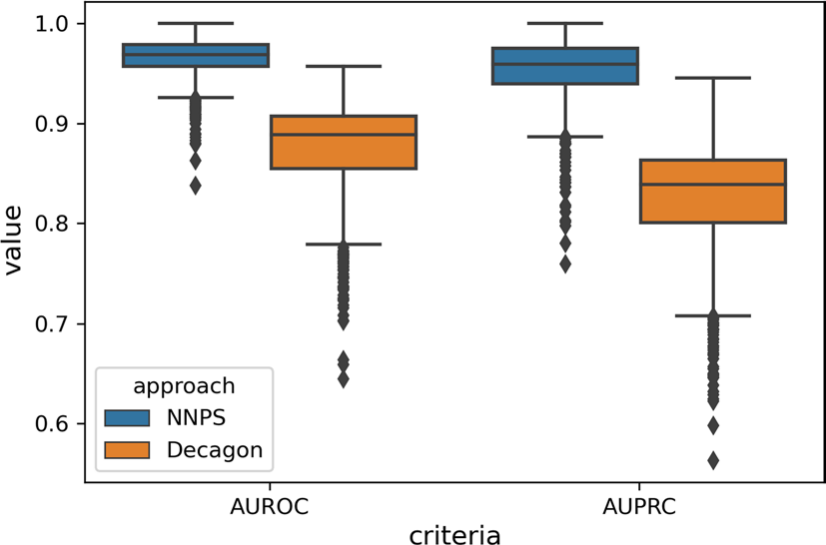
Boxplot of area under the receiver-operating characteristic (AUROC) and area under the precision–recall curve (AUPRC) values of all 964 side effects for NNPS and Decagon methods

In this section, the performance of NNPS is benchmarked against 5 well-known methods, Decagon, Concatenated drug features, Deep Walk, DEDICOM, and RESCAL, for 964 polypharmacy side effects. We adopt the 5-fold cross-validation for 50 iterations and use the average of the results to assess the performance of the NNPS method. The average of AUROC and AUPRC values of all methods for 964 polypharmacy side effects are presented in Table 4. Because only the source code and implementation of Decagon are available, we execute 5-fold cross-validation for 50 iterations for the Decagon method and see that the obtained results are very similar to the reported results of the Decagon method in [16]. In Table 4, we mention the average of the obtained results for the Decagon method and the reported performances of other methods that we do not have their source code by using Table 2 in [16]. According to Table 4, NNPS achieves the improvement 9.2% and 12.8% against Decagon which is the best algorithm among other well-known methods in terms of AUROC and AUPRC, respectively. To compare the results of NNPS more precisely, we compare it to the results of the Decagon with more details and by some more criteria. Figure 4 illustrates the boxplots of AUROC and AUPRC criteria for 964 polypharmacy side effects resulted by NNPS and Decagon methods, respectively. As shown in Fig. 4, it can be concluded that the median of the AUROC and AUPRC criteria related to NNPS are much higher than the median of the AUROC and AUPRC criteria related to the Decagon method, and the range of variation of the AUROC and AUPRC criteria for NNPS method are less than the range of variation of the AUROC and AUPRC criteria for the Decagon method which is the evidence of good performance of NNPS.

**Table 4:** The average of Area under ROC curve (AUROC), area under precision–recall curve (AUPRC) for 964 polypharmacy side effects prediction

Method	AUROC	AUPRC
NNPS	**0.966**	**0.953**
Decagon	0.874	0.825
Concatenated drug features	0.793	0.764
DeepWalk	0.761	0.737
DEDICOM	0.705	0.637
RESCAL	0.693	0.613

Bold numbers show the best performance for each criteria

For more evaluation, the best thresholds that have produced the best results for each polypharmacy side effects based on F-score values for NNPS and Decagon methods are detected and the results of NNPS and Decagon based on F-score, Accuracy (ACC), and Matthews Correlation Coefficient (MCC) are compared. Table 5 reports True Positive (TP), False Positive (FP), True Negative (TN), False Negative (FN), Precision, Recall, F-score, ACC and MCC of these two methods for all 964 side effects. According to Table 5, NNPS outperforms about 8.6%, 10.3%, and 18.1% against Decagon based on F-score, ACC, and MCC criteria, respectively.

**Table 5:** The average of the best results for NNPS and Decagon methods for 964 side effects

Method	TP	FP	TN	FN	Precision	Recall	F-score	ACC	MCC
NNPS	105,153	**12,013**	95,955	**2608**	**0.901**	**0.976**	**0.936**	**0.934**	**0.872**
Decagon	**208,963**	54,855	**163,988**	9880	0.771	0.950	0.850	0.831	0.685

Bold numbers show the best performance for each criteria

**Fig. 5:**
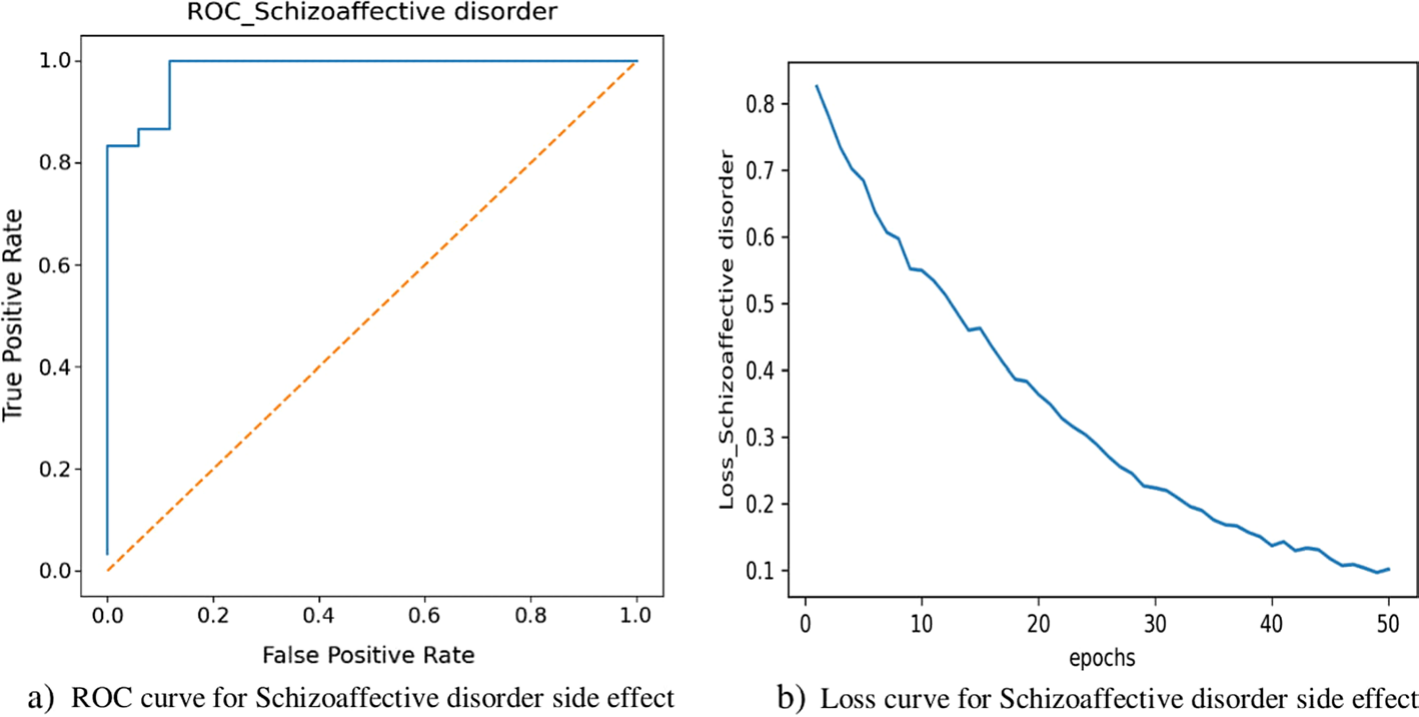
Receiver-Operating Characteristic (ROC) curve (part **a**) and loss curve (part **b**) of Schizoaffective disorder polypharmacy side effect for 50 epochs

### Evaluation of feature selection, aggregation, and train/test set sizes

In this part, to show the significance of the PCA algorithm for dimension reduction, we compare the results of NNPS by using the low variance filter and autoencoder techniques as two another feature selection methods. We use these two techniques to reduce the mono side effects and drug–protein interaction matrices features to 503 and 22 features, respectively. In Table 6, the results of NNPS with both dimension reduction techniques are presented. This table shows that the performance of the NNPS method is higher when PCA technique was used. Also, we adopt two operators (i.e., summation and concatenation) to aggregate the feature vectors of two drugs into one feature vector for representing the drug-drug pairs in neural network architecture. As shown in Table 7, the summation operator achieves better results with respect to the results of NNPS when we concatenate the feature vectors of two drugs as features for feeding the neural network. We train the NNPS method with two different size of train, validation, and test sets, and represent the results in Table 8. This table shows that the performance of the NNPS method has very little reduction by decreasing the size of the train set which is evidence of the advantage of the method. Finally, we compared the performance of our method to four well-known machine learning algorithms using AUROC and AUPRC. The average results of these methods for all 964 polypharmacy side effects are shown in Table 9. According to the values in the Table 9, NNPS has the best performance among all methods.

**Table 6:** The results for three dimension reduction techniques for 964 side effects

Dimension reduction techniques	TP	FP	TN	FN	AUROC	AUPRC	Precision	Recall	F-score	ACC	MCC
PCA	**105,153**	**12,013**	**95,955**	**2608**	**0.966**	**0.953**	**0.901**	**0.976**	**0.936**	**0.934**	**0.872**
Low variance filter	104,830	12,667	95,119	3113	0.963	0.952	0.899	0.972	0.933	0.931	0.866
Autoencoder	104,498	13,087	94,827	3317	0.963	0.950	0.897	0.971	0.931	0.929	0.862

Bold numbers show the best performance for each criteria

**Table 7:** Results of two feature aggregation operators for 964 side effects

Operators	thr	TP	FP	TN	FN	AUROC	AUPRC	Precision	Recall	F-score	ACC	MCC
Summation	0.530	**105,153**	**12,013**	**95,955**	**2608**	**0.966**	**0.953**	**0.901**	**0.976**	**0.936**	**0.934**	**0.872**
Concatenation	0.503	102,005	17,591	90,290	5843	0.943	0.930	0.867	0.947	0.904	0.900	0.806

Bold numbers show the best performance for each criteria

**Table 8:** The results of NNPS method with different size of Training set (Tr set), Validation and Test sets (VT sets) of dataset

Method	Tr set (%)	VT sets (%)	thr	TP	FP	TN	FN	AUROC	AUPRC	Precision	Recall	F-score	ACC	MCC
NNPS	60	20	0.508	416,152	56,575	375,325	16,221	0.961	0.948	0.885	0.961	0.921	0.918	0.839
NNPS	70	15	0.522	313,947	39,974	283,681	10,456	0.964	0.951	0.892	0.967	0.928	0.925	0.854

**Table 9:** Results of different machine learning methods

Method	AUROC	AUPRC	Running time
NNPS	**0.966**	**0.953**	**8 h, 40 min**
LassoCV	0.930	0.917	11 h, 30 min
SVM	0.871	0.813	1 day
Random forest	0.797	0.742	1 h, 30 min
KNN	0.746	0.692	12 h

Bold numbers show the best performance for each criteria

### Time complexity

Between the previous methods, only the source code and implementation of Decagon are available. So, we can only compare the time complexity of NNPS to Decagon method. The time of NNPS is about 8 h (Linux (Ubuntu 16.04), 15 CPUs, Intel Xeon(R) 2.00 GHz) on DPIs and DDIs datasets and is therefore noticeably faster than Decagon which requires 15 days for 5-fold cross-validation on a single GTX1080Ti graphic card. This decreased training time in NNPS that stems from the simplicity and efficiency of this model, is one of the main advantages of NNPS which can further be generalized to other purposes and datasets as well.

## Discussion and conclusion

**Fig. 6:**
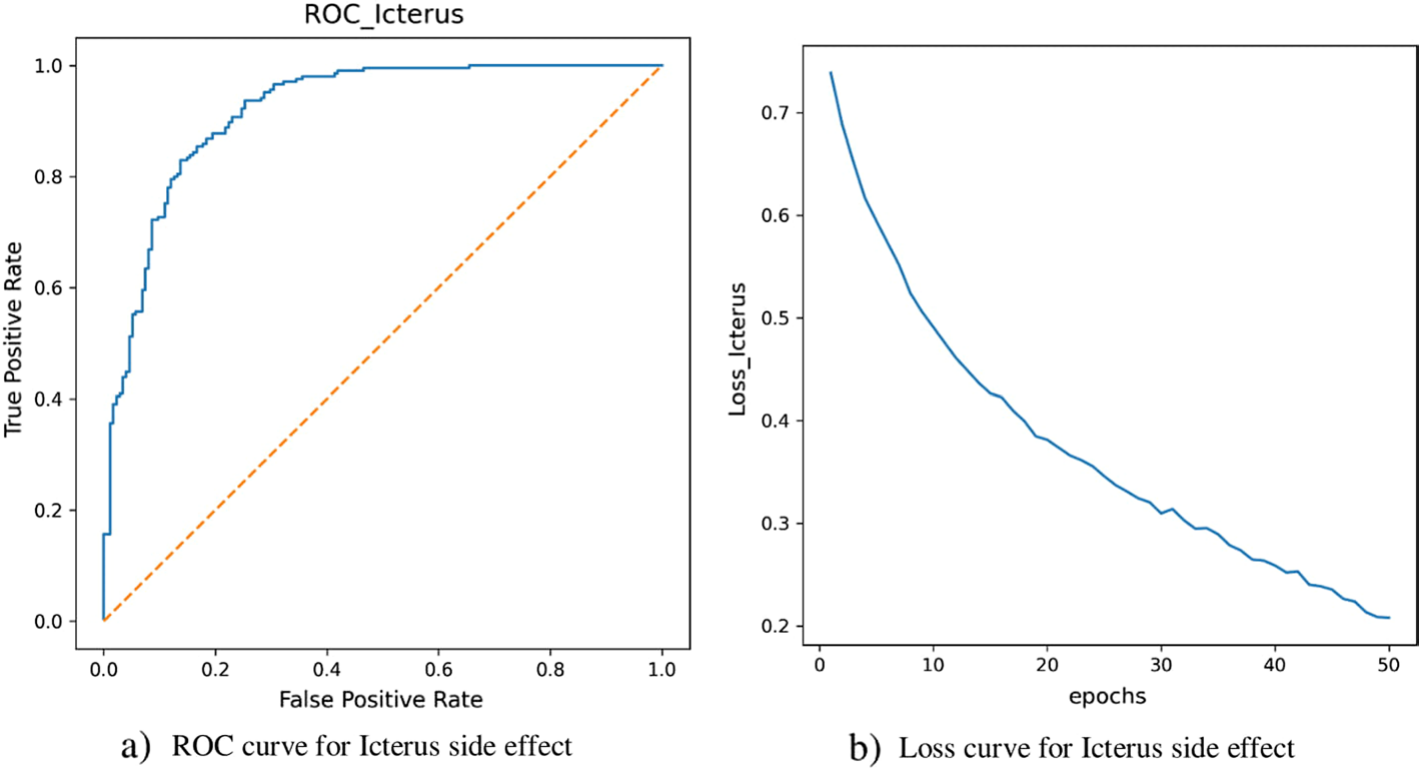
Receiver-Operating Characteristic (ROC) curve (part **a**) and loss curve (part **b**) of Icterus polypharmacy side effect for 50 epochs

Due to the enormous number of drug combinations, screening all possible pairs to achieve polypharmacy side effects are unfeasible in terms of cost and time. On the other hand, understanding the side effects of DDIs is an essential step in drug development and drug co-administration. So, some computational methods are developed for predicting polypharmacy side effects. The lately approach in this task (Decagon method) predicts the performance of polypharmacy side effects up to 0.874 and 0.825 in terms of accuracy on AUROC and AUPRC, respectively. In this study, we consider a neural network architecture with novel feature vectors. In NNPS method, each drug represents by a feature vector based on mono side effects and drug–protein interactions, and to decrease the method complexity, the PCA is used for dimension reduction of feature vectors. For a given drug pair, the corresponding drug feature vectors are summed to train the neural network for predicting polypharmacy side effects. The superior performance of NNPS occurs for two reasons. The first main reason is the novel feature vectors that are obtained by the dimension reduction techniques. The second reason is chosen a simple neural network architecture. We can see NNPS achieves excellent accuracy on the polypharmacy side effects prediction task that are shown in Additional file 1 and Table 10. We have provided 10 best and worst performance polypharmacy side effects based on AUROC and AUPRC in both NNPS and Decagon methods. The results can be found in Additional file 1: Tables S2–S7. These tables belong to the results of NNPS and Decagon which show that the performance of the NNPS method is better than the performance of the Decagon method. Figure 5 part (a) shows the ROC curve for Schizoaffective disorder side effect (one of the best performances of NNPS). Part (b) of Fig. 5 illustrates the loss curve of model for different epochs. Similarly, Fig. 6 part (a) and (b) show the ROC and Loss curves for NNPS related to Icterus side effect, one of the worst performances of NNPS, respectively. As shown by these figures, NNPS works well for each side effect alone and is acceptable with respect to the loss values for epoch 50. Among side effects with the best performance in NNPS, five important side effects that can lead to death or serious complications are selected [54–58]. The performance results in NNPS and Decagon methods and the literature evidence for supporting these dangerous side effects are collected in Table 10. According to Table 10, the performances of dangerous polypharmacy side effects in NNPS on AUROC have values of 1.0, but in Decagon are located between 0.791 and 0.936. Also, we can see that on AUPRC the NNPS method have values of 1.0 but the Decagon performances are between 0.789 and 0.911. The finding of this tables show that in dangerous side effects, the performance of NNPS is higher than the performance of Decagon, and the NNPS is an effective approach for predicting polypharmacy side effects especially in order to detect dangerous side effects.

**Table 10:** Results of dangerous side effects in NNPS and Decagon on AUROC and AUPRC

Polypharmacy side effects	AUROC (NNPS)	AUROC (Decagon)	AUPRC (NNPS)	AUPRC (Decagon)	Evidence
Sarcoma	**1.0**	0.791	**1.0**	0.789	Serban et al. [54]
Carcinoma of the cervix	**1.0**	0.879	**1.0**	0.810	Arbyne et al. [55]
Malignant hypertension	**1.0**	0.906	**1.0**	0.858	Januszewicz et al. [56]
Epidural hematoma	**1.0**	0.936	**1.0**	0.906	Atci et al. [57]
Oophorectomy	**1.0**	0.917	**1.0**	0.911	Evans et al. [58]

Bold numbers show the best performance

In summary, the evaluation of the NNPS against five well-known methods, in terms of accuracy, complexity, and running time speed shows the performance of the presented method for an essential and challenging problem in pharmacology.

As for future work, we suggest adding the protein–protein interaction information to the model, as it plays a crucial role in many biological functions and may lead to more accurate results. Another avenue for research is to apply the proposed method to other datasets and compare their findings on the association of diseases and polypharmacy side effects with the current work.

## Supplementary Information

**Additional file 1.** Different hyperparameters values for 964 side effects of each model, and the results of 10 best and worst performance of polypharmacy side effects in NNPS and Decagon on AUROC and AUPRC. Bold numbers show the best performance for each criteria.

## Data Availability

Datasets and code for NNPS algorithm are freely accessible at https://github.com/raziyehmasumshah/NNPS.
