# Supplementary material for: A neural network-based method for polypharmacy side effects prediction
Source: BMC Bioinformatics. 2021 Jul 24;22:385. doi: 10.1186/s12859-021-04298-y (PMC8305591; doi:10.1186/s12859-021-04298-y)
Supplement: Supplementary file 1 — Additional file 1. Different hyperparameters values for 964 side effects of each model, and the results of 10 best and worst performance of polypharmacy side effects in NNPS and Decagon on AUROC and AUPRC. Bold numbers show the best performance for each criteria. [file 12859_2021_4298_MOESM1_ESM.pdf]

Supp Table 1: Different hyperparameter values for 964 side effect of each model

Method	No. hidden layers	No. neurons	Activation function	Dropout rate	Learning rate	Momentum
Model-1	1	300	relu, sigmoid	0.1	0.01	0.9
Model-2	2	300,200	relu,relu,sigmoid	0.1	0.01	0.9
Model-3	3	300,200,100	relu,relu,relu,sigmoid	0.1	0.01	0.9
Model-4	3	100,200,300	relu,relu,relu,sigmoid	0.1	0.01	0.9
Model-5	3	300,200,200	relu,relu,relu,sigmoid	0.1	0.01	0.9
Model-6	3	300,100,100	relu,relu,relu,sigmoid	0.1	0.01	0.9
Model-7	3	300,200,100	tanh,tanh,tanh,sigmoid	0.1	0.01	0.9
Model-8	3	300,200,100	sigmoid,sigmoid,sigmoid,sigmoid	0.3	0.01	0.9
Model-9	3	300,200,100	tanh,tanh,tanh,tanh	0.5	0.01	0.9
Model-10	3	300,200,200	relu,relu,relu,sigmoid	0.1	0.001	0.9
Model-11	3	300,200,200	relu,relu,relu,sigmoid	0.1	0.01	0.7

Supp Table 2: AUROC results of NNPS and Decagon on best performance side effects in NNPS

Best performance side effects in NNPS	AUROC (NNPS)	AUROC (Decagon)
Arachnoiditis	**1.0**	0.744
MPD	**1.0**	0.746
Sinus arrest	**1.0**	0.832
Colon neoplasm	**1.0**	0.826
Sarcoma	**1.0**	0.791
Carcinoma of the cervix	**1.0**	0.879
Pneumoconiosis	**1.0**	0.856
Malignant hypertension	**1.0**	0.906
Epidural hematoma	**1.0**	0.936
Oophorectomy	**1.0**	0.917

Supp Table 3: AUPRC results of NNPS and Decagon on best performance side effects in NNPS

Best performance side effects in NNPS	AUPRC (NNPS)	AUPRC (Decagon)
Arachnoiditis	**1.0**	0.694
MPD	**1.0**	0.685
Sinus arrest	**1.0**	0.776
Colon neoplasm	**1.0**	0.788
Sarcoma	**1.0**	0.789
Carcinoma of the cervix	**1.0**	0.810
Pneumoconiosis	**1.0**	0.834
Malignant hypertension	**1.0**	0.858
Epidural hematoma	**1.0**	0.906
Oophorectomy	**1.0**	0.911

Supp Table 4: AUPRC results of NNPS and Decagon on best performance side effects in Decagon

Best performance side effects in Decagon	AUPRC (Decagon)	AUPRC (NNPS)
Mumps	0.964	**0.981**
Carbuncle	0.949	**0.963**
Coccydynia	0.943	**0.995**
Tympanic membrane perforation	0.941	**1.0**
Dyshidrosis	0.938	**0.995**
Spondylosis	0.929	0.929
Schizoaffective disorder	0.919	**0.920**
Breast dysplasia	0.918	**0.999**
Ganglion	0.909	**0.999**
Uterine polyp	0.908	**1.0**

Supp Table 5: AUROC results of NNPS and Decagon on worst performance side effects in NNPS

Worst performance side effects in NNPS	AUROC (NNPS)	AUROC (Decagon)
Corneal abrasion	**0.913**	0.820
Eosinophilic pneumonia	**0.916**	0.854
Haemarthrosis	**0.886**	0.868
Aplasia pure red cell	**0.883**	0.848
Patent ductus arteriosis	**0.889**	0.708
Lung adenocarcinoma	**0.933**	0.724
Glomerulonephritis	**0.879**	0.842
Endocarditis	**0.919**	0.865
Transfusion reaction	**0.913**	0.723
Hypoglycaemia neonatal	**0.862**	0.903

Supp Table 6: AUPRC results of NNPS and Decagon on worst performance side effects in NNPS

Worst performance side effects in NNPS	AUPRC (NNPS)	AUPRC (Decagon)
Corneal abrasion	0.759	**0.783**
Eosinophilic pneumonia	0.780	**0.802**
Haemarthrosis	0.797	**0.813**
Aplasia pure red cell	**0.801**	0.793
Patent ductus arteriosis	**0.802**	0.649
Lung adenocarcinoma	**0.811**	0.720
Glomerulonephritis	**0.816**	0.739
Endocarditis	**0.818**	0.803
Transfusion reaction	**0.830**	0.716
Hypoglycaemia neonatal	0.835	**0.884**

Supp Table 7: AUPRC results of NNPS and Decagon on worst performance side effects in Decagon

Worst performance side effects in Decagon	AUPRC (Decagon)	AUPRC (NNPS)
Bleeding	0.679	**0.931**
Increased body temperature	0.680	**0.950**
Emesis	0.693	**0.943**
Renal disorder	0.694	**0.951**
Leucopenia	0.695	**0.898**
Diarrhea	0.705	**0.942**
Icterus	0.707	**0.936**
Nausea	0.711	**0.940**
Itch	0.712	**0.952**
Anaemia	0.712	**0.945**
